# AFM-Based Force Spectroscopy Guided by Recognition Imaging: A New Mode for Mapping and Studying Interaction Sites at Low Lateral Density

**DOI:** 10.3390/mps2010006

**Published:** 2019-01-08

**Authors:** Melanie Koehler, Anny Fis, Hermann J. Gruber, Peter Hinterdorfer

**Affiliations:** 1 Institute of Biophysics, Johannes Kepler University, 4020 Linz, Austria; melanie.kohler@uclouvain.be (M.K.); anny.fis@jku.at (A.F.); hermann.gruber@jku.at (H.J.G.); 2 Louvain Institute of Biomolecular Science and Technology, Université Catholique de Louvain, 1348 Louvain-La-Neuve, Belgium

**Keywords:** atomic force microscopy, single molecule force spectroscopy, recognition imaging, membrane, receptor–ligand interaction, energy landscape

## Abstract

Ligand binding to receptors is one of the most important regulatory elements in biology as it is the initiating step in signaling pathways and cascades. Thus, precisely localizing binding sites and measuring interaction forces between cognate receptor–ligand pairs leads to new insights into the molecular recognition involved in these processes. Here we present a detailed protocol about applying a technique, which combines atomic force microscopy (AFM)-based recognition imaging and force spectroscopy for studying the interaction between (membrane) receptors and ligands on the single molecule level. This method allows for the selection of a single receptor molecule reconstituted into a supported lipid membrane at low density, with the subsequent quantification of the receptor–ligand unbinding force. Based on AFM tapping mode, a cantilever tip carrying a ligand molecule is oscillated across a membrane. Topography and recognition images of reconstituted receptors are recorded simultaneously by analyzing the downward and upward parts of the oscillation, respectively. Functional receptor molecules are selected from the recognition image with nanometer resolution before the AFM is switched to the force spectroscopy mode, using positional feedback control. The combined mode allows for dynamic force probing on different pre-selected molecules. This strategy results in higher throughput when compared with force mapping. Applied to two different receptor–ligand pairs, we validated the presented new mode.

## 1. Introduction

Atomic force microscopy (AFM) is one of the most versatile microscopic tools due to its capability to provide not only topographical images, but also to probe and characterize interactions on the single molecular level [1]. Information on unbinding forces, kinetic rate constants, bond life times, and energy landscapes can be obtained. The technique of AFM-based single molecule force spectroscopy (SMFS) is based on the following principle: A functionalized AFM cantilever is approached towards and subsequently retracted from the sample surface at constant velocity in the z-direction at fixed x–y coordinates. The cantilever’s deflection is continuously monitored to record the interaction force, which results in a so-called force-distance cycle (FDC). High densities and homogeneous distributions of receptor molecules are required for performing a successful force spectroscopy experiment, as the tip is “blindly” moved in the z-direction. Such conditions are not always easily applicable, in particular when the molecules of interest are membrane proteins. Only low reconstitution efficiencies are often obtained for protein integration into an artificial lipid membrane. To overcome this issue, an AFM-technique known as “force volume (FV)” was initially proposed by Cleveland et al. [2] and further used in pioneering studies by Heinz and Hoh [3] and Gaub et al. [4]. In this mode, the cantilever records one or more FDCs at every lateral position, as it moves across the surface. The main drawback of this handy technique is the acquisition time. The required time for obtaining such a map is directly linked to the selected parameters (image size, pixel resolution, number of FDCs per pixel, FDC sweep time, etc.) and can range from minutes to several hours, when a high-resolution map is needed. Based on the principle of FV, a new mode was developed aiming to overcome the above-mentioned time limitations, i.e., peak force tapping (PFT). Despite the fact that PFT was successfully applied on a variety of biological samples, ranging from membrane proteins [5] to entire cells [6], for high-resolution maps, a few hours are still required.

In 2017, a combination of two techniques, simultaneous topographical and recognition (TREC) imaging and SMFS was suggested by Koehler et al. [7]. TREC and SMFS are two AFM techniques that ideally complement each other. Topographical and recognition imaging is an imaging technique that provides information on receptor binding sites and SMFS can fully characterize a molecular interaction by quantitatively determining its energetic and kinetic parameters. The advantage of TREC-driven force spectroscopy is that a single functional molecule can be selected and targeted for force spectroscopy.

We describe here a detailed protocol of performing AFM-based force spectroscopy guided by recognition imaging in order to map and characterize binding sites of membrane proteins reconstituted into lipid bilayers. 

## 2. Experimental Design

**Figure 1:**
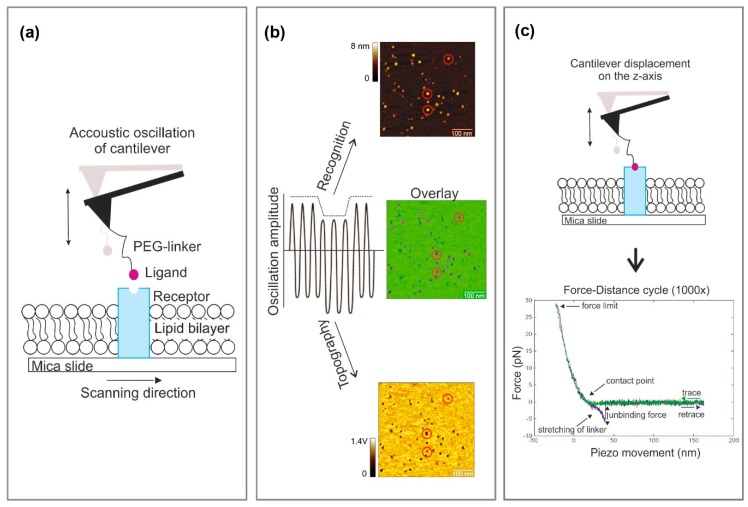
Experimental setup of atomic force microscopy (AFM)-based force spectroscopy guided by recognition imaging. (**a**) Receptors reconstituted into a planar lipid bilayer spread on a mica surface. The cantilever is oscillated by acoustical excitation over the sample surface. (**b**) Simultaneous topographical and recognition (TREC) imaging. The oscillation amplitude is split into two parts. The upper part is taken to construct the recognition image, whereas the lower part is used for the topography image. Specific interaction of the tip-bound ligand with the receptor on the surface leads to a recognition event that is visible as a black dot in the recognition image (red circles). The overlaid image reveals a superposition of the recognition map on the topographical image. (**c**) The cantilever is placed over the functional receptor (i.e., molecules that generated recognition signal (red circles)) and the mode is switched to force spectroscopy. In this mode, the cantilever is repeatedly approached and retracted from the sample surface, resulting in continuous recordings of force–distance cycles. A typical ligand-receptor unbinding event arising from force-induced ligand/receptor dissociation appears as a parabolic-shaped downward signal. Specific unbinding lengths above 10 nm can be attributed to the elasticity of the receptors and the lipid membrane. PEG: Polyethylene glycol.

Based on AFM tapping mode in liquid [8,9], a cantilever tip carrying a ligand molecule is oscillated across a membrane. Topography and recognition images of reconstituted receptors are recorded simultaneously by analyzing the downward and upward parts of the oscillation, respectively. Functional receptor molecules are then selected from the recognition image with nanometer resolution before the AFM is switched to the force spectroscopy mode, using positional feedback control [7]. The combined mode is an optimal tool for dynamic force probing on different pre-selected molecules, resulting in higher throughput when compared with force mapping. Moreover, the dynamics of force loading can be varied to elucidate the binding dynamics and map the interaction energy landscape [10]. A general functional principle of this technique is illustrated in Figure 1. 

Combining the techniques of TREC imaging [11] and force spectroscopy [12] faces three major challenges: (i) A common technical issue during AFM measurements is thermal drift, which can lead to the loss of the protein position between two imaging scans or during force spectroscopy experiments. This drawback can be significantly improved by using a closed loop scanner (N9524A (Keysight, Santa Rosa, CA, USA): a multipurpose scanner with different nose cone cantilever modules, for acoustic or magnetic excitation), in which inductive sensors detect the deviations from the ideal movement and apply appropriate corrections to the piezo drive signal. (ii) It is mandatory to use the same cantilever for the entire combined TREC and force spectroscopy experiment. For an optimal force sensitivity a rather soft spring is required (spring constant 0.01–0.03 N/m) in conventional force spectroscopy [13], whereas for high-resolution dynamic imaging a stiffer spring (0.14–0.30 N/m) is preferred [11]. By identifying an acceptable compromise to satisfy both needs, a silicon nitride tip with a nominal spring constant of 0.03 N/m (MSNL levers (Bruker AFM Probes, Camarillo, CA, USA)) was used. Since such tips are not available with a magnetic coating (the standard for the magnetic excitation commonly used in TREC [11]), an acoustic excitation of the cantilever was applied instead. We thus optimized an alternative TREC mode for cantilevers with a small spring constant. (iii) In order to optimize the remaining drift with the usage of the closed loop scanner, constant environmental conditions were ensured. We strictly maintained a constant lab temperature (room temperature) and started the measurements only when the system (sample, measurement buffer, and cantilever) appeared equilibrated.

In the rare event of loss of unbinding signals during force spectroscopy, the microscope was switched back to the recognition imaging mode and the cantilever was readjusted to the protein molecule under investigation. However, by using a positional feedback control together with a careful thermal stabilization protocol, the measurements were reasonably stable for at least 2 h without loss of the binding site.

The procedures described in this protocol detail how this combined mode can be successfully conducted. In the first part (Section 3.1), important references are provided on how proteoliposomes with containing membrane proteins can be prepared. The timing procedure depends on the chosen lipid composition and reconstituted protein, and can take up several days. The second part (Section 3.2) describes how the lipid bilayer with the incorporated receptors is formed on a surface for conducting the AFM experiments (time: 15 min up to ~2 h). In the third part (Section 3.3), the AFM cantilever is functionalized with a cognitive ligand by utilizing a heterobifunctional crosslinker that binds to both the amino-derivatized tip and the free amino-groups of the ligand. This procedure takes 2–3 days. The fourth part (Section 3.4) describes detailed procedures for application of this combined mode to characterize interaction sites at low lateral density. An additional chapter (4) reports the expected results and further applications. The proof-of-concept was shown for the quantitative characterization of the binding mechanism between mitochondrial membrane uncoupling protein 1 (UCP1) and its inhibitor adenosine triphosphate (ATP). Here the binding dynamics as well as the interaction energy landscape was elucidated [7,14] (Section 4.1). In the second example, the interaction between the bacterial translocation channel SecYEG and the cytoplasmic motor-protein SecA was characterized. The AFM-based force spectroscopy guided by recognition imaging was employed in order to localize the SecYEG complexes and map SecA binding sites on suspended lipid bilayers. Subsequently, the interaction forces between SecYEG and SecA interaction were characterized. Finally, Section 4.3 provides detailed information on how the data were analyzed and interpreted. 

### 2.1. Materials

- Acetal–PEG_27_–NHS (available by purchase from H. Gruber, Johannes Kepler University Linz [15], Linz, Austria). Alternatively, this linker can be synthesized according to Reference [16]. 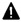 **CRITICAL STEP** We recommend purchasing monodisperse polyethylene glycol (PEG) linkers from this source for two reasons. First, the benzaldehyde function is protected by an acetal-group for the binding to amino-functionalized tips via the activated carboxyl functionality of the N-hydroxysuccinimide (NHS)-group. This elegantly precludes the possibility of bivalent interaction of the benzaldehyde function with adjacent amino-groups on the tip surface. Second, the linker is supplied in high-purity 1-mg aliquots ideally suited for use in this protocol.
- Dimethylsulfoxide anhydrous (>99.9%, (CH_3_)_2_SO (DMSO); Sigma–Aldrich, St. Louis, MO, USA, cat. no. 41640) 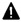 **CAUTION** DMSO is harmful upon inhalation and skin absorption. Wear appropriate gloves.
- Sodium cyanoborohydride (NaCNBH_3_; Sigma–Aldrich, cat. no. 156159) 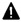 **CAUTION** NaCNBH_3_ is highly toxic. Wear gloves and handle carefully.
- Chloroform (>99.9%, CHCl_3_; Sigma–Aldrich, cat. no. 288306) 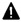 **CAUTION** Chloroform is highly volatile and harmful upon inhalation and skin absorption. Wear appropriate gloves and work under a fume hood. Handle only in glass vessels and glass dishes covered with a lid.
- Ethanol, absolute (>99.9%, C_2_H_5_OH; Sigma–Aldrich, cat. no. 24102).
- (3-Aminopropyl)triethoxysilane (APTES, 99%, Sigma–Aldrich, cat. no. 440140). Distill at low temperature and store under argon in sealed crimp vials over silica gel at −20 °C to avoid polymerization.
- Ethanolamine hydrochloride (H_2_NC_2_H_4_OH; Sigma–Aldrich, cat. no. E6133).
- 0.1 mM NaOH solution (in water, Sigma–Aldrich, cat. no. 43617).
- Molecular sieves, 3 Å (beads, 8–12 mesh; Sigma–Aldrich, cat. no. 208574).
- Triethylamine >99.5%, (C_2_H_5_)_3_N; Sigma–Aldrich, cat. no. 471283) 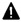 **CAUTION** Triethylamine is harmful upon inhalation and skin absorption, and is volatile. Wear appropriate gloves and work under a fume hood. Store under argon and in the dark to avoid amine oxidation.
- Milli-Q water (Merck Millipore, Burlington, MA, USA).
- Citric acid (Sigma–Aldrich, cat. no. 251275).
- Stock of membrane proteins, reconstituted into proteoliposomes. UCP1 and SecYEG for the examples shown here.
- Ethylenediamine (EDA) derivates of the purine nucleotides (EDA-ATP, EDA-ADP and EDA-AMP) (BioLog, Bremen, Germany, cat. no. A072, A118) or SecA protein for tip coupling.
- Na_2_SO_4_ (Sigma-Aldrich, cat. no. 238597).
- (NH_4_)_2_HPO_4_ (Sigma–Aldrich, cat. no. 215996).
- 2-(N-morpholino)ethanesulfonic acid (MES) (Sigma–Aldrich, cat. no. M3671).
- Tris (Sigma–Aldrich, cat. no. 252859).
- Ethylene glycol-bis(β-aminoethyl ether)-N,N,N′,N′-tetraacetic acid (EGTA) (Sigma–Aldrich, cat. no. E3889).
- HEPES (Sigma–Aldrich, cat. no. H3375).
- HEPES potassium salt (K-HEPES) (Sigma–Aldrich, cat. no. H0527).
- Sodium Chloride (NaCl) (Sigma–Aldrich, cat. no. S7653).
- Calcium Chloride (CaCl_2_) (Sigma–Aldrich, cat. no. C1016).
- Magnesium Chloride (MgCl_2_) (Sigma–Aldrich, cat. no. M8266)
- Potassium Chloride (KCl) (Sigma–Aldrich, cat. no. P9333)
- Muscovite mica sheets (V-1 quality, Electron Microscopy Science, Hatfield, PA, USA, cat. no. 71855)

### 2.2. Equipment

- Keysight 5500 AFM setup (or higher) equipped with PicoPlus box.
- Closed-loop scanner (N9524A, Keysight).
- AFM tapping nose cone for acoustic excitation (Keysight).
- MSNL-lever (Bruker AFM Probes).
- Inverted optical microscope.
- Upright benchtop optical microscope for examining cantilevers and chips.
- Active vibration isolation table.
- Acoustic enclosure for the AFM.
- AFM flow-through liquid cell.
- N_2_ gas bottle for drying cantilever chips.
- Argon gas bottle for storing cantilever chips and chemicals properly without contamination.
- pH meter.
- Glass Pasteur pipettes and rubber bulbs for pH adjustment.
- Analytical balance.
- Fume hood.
- Vacuum pump.
- Vacuum desiccator.
- Oven.
- Plastic and glass Petri dishes (15 mm) with lid for tip functionalization and surface chemistry.
- Tweezer.
- Mechanical pipettors.
- Pipette tips.
- Hot plate stirrer.
- Magnetic stir bars.
- Glass beakers and Erlenmeyer flasks.
- 4-cm glass crystallization dishes (Sigma–Aldrich, cat. no. BR455701).
- Teflon block (3 × 1 × 0.5 cm).
- Teflon reaction chamber (2 × 2 × 1.5 cm block indented with a cylindrical chamber 1-cm deep and 1.5 cm in diameter).
- 12-well plates (Sigma–Aldrich, cat. no. CLS3513).
- Vortex.
- 50 µL and 500 µL Hamilton glass syringe.
- Gwyddion Analysis software (v. 2.49) or any other analysis software for scanning probe microscopy (SPM) images, such as Pico Image (Keysight) or ImageJ.
- Origin (Origin 2016, Origin) or any other scientific graphing and data analysis software.
- Matlab (2014 or higher) + kspec19 analysis routine (provided by Keysight).

## 3. Procedure

### 3.1. Proteoliposome Preparation and Protein Reconstitution. Time to Completion: Several Days, Depending on the Studied Biomolecule and Applied Protocol for Preparation

Depending on the desired protein, which were to be reconstituted into proteoliposomes, different protocols were available [17,18]. For the examples shown here (see Section 4 for expected results), the following protocols can be consulted:

- UCP1 in *Escherichia coli* polar lipid extract (PLE): Hilse et al. [19] and Rupprecht et al. [20].
- SecYEG in PLE: Knyazev et al. [21,22].

### 3.2. Surface Chemistry. Time to Completion: 15 min up to ~2 h, Depending on the Studied Biomolecule

1. Prepare a freshly cleaved mica by removing the upper layer of the quartz-like material with a commercial tape.
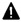
**CRITICAL STEP** Make sure to remove a closed layer, to generate an ultra-flat and clean surface for incubation of the protein-containing proteoliposomes.
2. Place the freshly cleaved mica on the AFM sample plate and mount a (flow-through) fluid cell.
3. Dilute the proteoliposome solution to a final concentration of 1 mg/mL lipid concentration with assay buffer.
4. After short vortexing, pipet the proteoliposome solution into the fluid cell and onto the clean and flat mica sheet.
5. Depending on the lipid composition and the protein, let the sample incubate for 10 min up to 2 h at room temperature (RT). After that, lipid bilayer batches containing the protein of choice should be formed.
  **OPTIONAL STEP** Depending on the lipid composition (and their phase transition temperatures), you may raise the temperature up to 60 °C during the incubation.
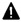
**CRITICAL STEP** Make sure to always keep the sample moist to prevent evaporation of the liquid during the incubation.
6. Wash the sample thoroughly with assay buffer and add 500–600 µL assay buffer to the fluid cell for AFM measurements.

### 3.3. AFM Tip Functionalization. Time to Completion: 2–3 Days, Depending on the Chosen Aminofunctionalization Procedure 

The most common functionalization method is tethering the ligand on a silicon/silicon nitride AFM tip in a multiple-step crosslinking procedure, including the creation of reactive amino groups on the chemically inert tip surface (Section 3.3.1), followed by the covalent binding of the crosslinker (Section 3.3.2), and finally, coupling of the sensor molecule (Section 3.3.3). The inert water soluble PEG-crosslinker was designed as hetero-bifunctional [23], with one end being an *N*-hydroxysuccinimide (NHS) group to bind free amino groups on the AFM tip and the other end specifically designed to react with the desired ligand for coupling. The latter is available with different reactive molecules, such as an acetal-group to couple lysine residues (NH_2_), a malemide-group to couple thiol (SH)-containing molecules or a tris-nitrilotriacetic acid (NTA) group to bind His_6_-tagged proteins. A general overview can be found in Reference [15]. 

For all tip functionalization procedures, the ligand density on the tip is adjusted such that on average only one ligand has access to the receptors on the surfaces. Therefore, our tip functionalization design is also apt for single molecule studies at high receptor densities, see H. Gruber et al. [15]. 

#### 3.3.1. Aminofunctionalization of the AFM Tip

In the following, two commonly used methods for aminofunctionalization of an AFM tip (or sample substrates) are presented according to Reference [15,24,25]. While method A is more solid and leads to a very homogenous meshwork of amino groups, it requires some chemical expertise and experience of the operator due to a reaction step in the gas phase and the volatility of both APTES and triethylamine. Moreover, APTES silanization is very critical with respect to the freshness of APTES (hydrolyzes very fast in air) and exclusion of air. For unexperienced users, method B in liquid phase is highly recommended. Both methods are fully satisfactory with respect to the attachment of ligands single molecule force spectroscopy experiments. Parallel macroscopic assays on silicon nitride chips showed that APTES appeared to yield a slightly lower surface density of amino groups [25]. 

Method A, with APTES in the gas phase.

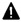
**CRITICAL STEP** Perform the whole procedure in a well-ventilated hood.
2. Wash cantilevers in chloroform (3 × 5 min), dry with nitrogen gas, continue with the next step.
3. Flush desiccator chamber (5 L) with argon gas (through the narrow opening in the lid).
4. Place a tray (caps from 1.5 mL plastic Eppendorf tubes) with 30 μL APTES and another tray with 10 μL triethylamine in the desiccator.
5. Place the cantilevers in the desiccator close to the two trays. Close the lid, incubate for 2 h.
6. Remove the trays with APTES and triethylamine, flush desiccator with argon. Incubate the tips in argon for at least 2 days (“curing”).
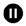
**PAUSE STEP** Store under argon in a dust box for up to 3 weeks (preferably <1 week), if they are not used immediately.

Method B, with Ethanolamine Hydrochloride in DMSO.

1. Wash cantilevers in chloroform (3 × 5 min), dry with nitrogen gas, continue with the next step.
2. Dissolve 3.3 g ethanolamine hydrochloride in 6.6 mL DMSO, cover with lid, heat to ~70 °C for complete dissolution, let cool to room temperature. In addition, a magnetic stirrer can be added to the solution to speed up the dissolving process.
3. Immerse a Teflon block for the cantilevers in the center of the dish and add 3–4 Å molecular sieves beads to cover the surrounding area (~25% of the total volume of the liquid).
4. Put the dish into a desiccator (or vacuum chamber) and degas the solution and the molecular sieves by applying an aspirator vacuum (or similar) for ~30 min.
5. Place cantilevers on the Teflon block, cover with lid, incubate at room temperature overnight.
6. Wash cantilevers in DMSO (3 × 1 min) and ethanol (3 × 1 min).
7. Dry with a gentle stream of nitrogen or argon gas.
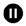
**PAUSE STEP** Store under argon in a dust box for up to 3 weeks (preferably <1 week), if they are not used immediately.

#### 3.3.2. NHS–PEG_27_–Acetal Coupling

1. Dissolve 1 portion of Acetal–PEG_27_–NHS (1 mg) in chloroform (0.5 mL), transfer the solution into the reaction chamber, add triethylamine (30 μL) and mix by pipetting.
2. Immediately place cantilever(s) in the reaction chamber, cover the chamber and incubate for 2 h.
3. Wash with chloroform (3 × 10 min) and dry with nitrogen or argon gas.
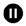
**PAUSE STEP** Store cantilever(s) for up to several months under argon, or continue with next step (Section 3.3.3).

#### 3.3.3. Coupling of Interacting, Corresponding Biomolecule to the Free Acetal-End

1. Immerse the cantilevers functionalized with NHS–PEG_27_–Acetal cross linker for 10 min in 1% (*w*/*v*) citric acid (in water).
2. Wash the cantilevers in Milli-Q water (3 × 5 min) and dry with nitrogen or argon gas.
3. Freshly prepare a 1 M solution of sodium cyanoborohydride (with 20 mM NaOH) from the following components: 13 mg NaCNBH_3_, 20 μL 100 mM NaOH, and 180 μL Milli-Q water.
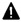
**CRITICAL STEP** NaCNBH_3_ is highly toxic. Wear gloves and handle with care.
4. Place the AFM chips in a radial arrangement, with the tips in the center and facing upwards, on Parafilm in a polystyrene Petri dish.
5. Pipet 100 μL protein solution (1–2 μM) carefully onto the tip of each cantilever so that they are extended into the protein drop. In the first example shown here, an EDA derivate of different purine nucleotides was coupled to the AFM tip to study their interaction with uncoupling proteins. In the second example, the ATP motor-protein SecA was attached onto the AFM tip in order to investigate its interaction with the bacterial translocon SecYEG.
6. Add 2 μL of the freshly prepared 1 M NaCNBH_3_ stock solution to the protein solution droplet, mix carefully by pipetting, cover with a lid, and incubate for 1 h.
7. **OPTIONAL STEP** Dissolve 3 pellets NaOH in 500 mL water, add the unused NaCNBH_3_ solution, mix, wait overnight, pour into the drain, and flush with tap water.
8. To passivate unreached aldehyde groups, add 5 μL of ethanolamine solution (1 M, pH 8.0) to the drop on the cantilever(s), mix cautiously by pipetting, cover with a lid, and incubate for 10 min.
9. Wash in PBS or any other buffer of choice (3 × 5 min).
10. Mount cantilever in AFM setup if used immediately for experiments.
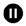
**PAUSE STEP** If not used immediately, store cantilever in a 24-well plate under PBS at 4 °C for 1–2 weeks (depending on the stability of the conjugated biomolecule).
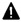
**CRITICAL STEP** Transfer the functionalized AFM cantilevers to PBS buffer and then to the AFM always very rapidly so that they do not dry (within 20 s). Otherwise, the conjugated biomolecule could start to denaturate and lose its bio-functionality.

### 3.4. AFM Measurement. Time to Completion: ~4 h up to 1 Day, Depending on the Studied Biomolecule and the Desired Data Throughput

1. Switch on the AFM equipped with a PicoPlus 5500 box (Keysight Technologies) and the AFM controlling software (PicoView, usable versions starting from 1.12 until 1.20).
2. Switch on the inverted optical microscope and the related controlling software.
3. Mount the AFM liquid cell with the sample on the AFM stage from the bottom.
4. Mount the AFM tip derivatized with the corresponding, interacting molecule onto the cantilever holder (tapping nose cone), slot the holder into the closed-loop scanner and assemble everything onto the AFM stage from the top.
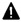
**CRITICAL STEP** Ensure that the Z stepper motors are sufficiently retracted to prevent crashing the cantilever into the bottom to the fluid cell and onto the sample.
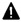
**CRITICAL STEP** Perform these steps rapidly. A drop of the assay buffer should always remain on the cantilever chip during transfer and should not be allowed to dry out.
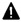
**CRITICAL STEP** This experiment works only with closed-loop scanners (e.g., N952A, Keysight Technologies) and the tip acoustically excited. Make sure to connect the cable for the closed-loop scanner on the AFM stage and switch on the closed-loop option in the software. Moreover, use the right nose cone (for acoustic excitation, “tapping nose”).
5. Align the laser spot at the free end of the cantilever and maximize the sum into the photodetector.
6. Bring the cantilever close to contact with the sample as follows: Using a wide-field imaging mode and the manual controls, locate the shadow of the cantilever by adjusting the AFM head position until the cantilever is directly in the light path. Using the AFM software, slowly drive the Z stepper motors of the AFM head down until the cantilever is within the focal range of the microscope objective and bring it into focus. It might be necessary to make fine adjustments in the position of the laser spot at this time.
7. Allow 15 min up to 1 h for the system to equilibrate, until the vertical offset of the laser spot on the photodiode no longer drifts. Try to keep the lab temperature constant during the whole experiment.
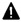
**CRITICAL STEP** Perform this step very thoughtfully and considerate. A stable system with as less thermal drift (in x–y directions) as possible is very important for the upcoming experimental steps.

#### 3.4.1. TREC Imaging: Protein Distribution and Functionality

**Figure 2:**
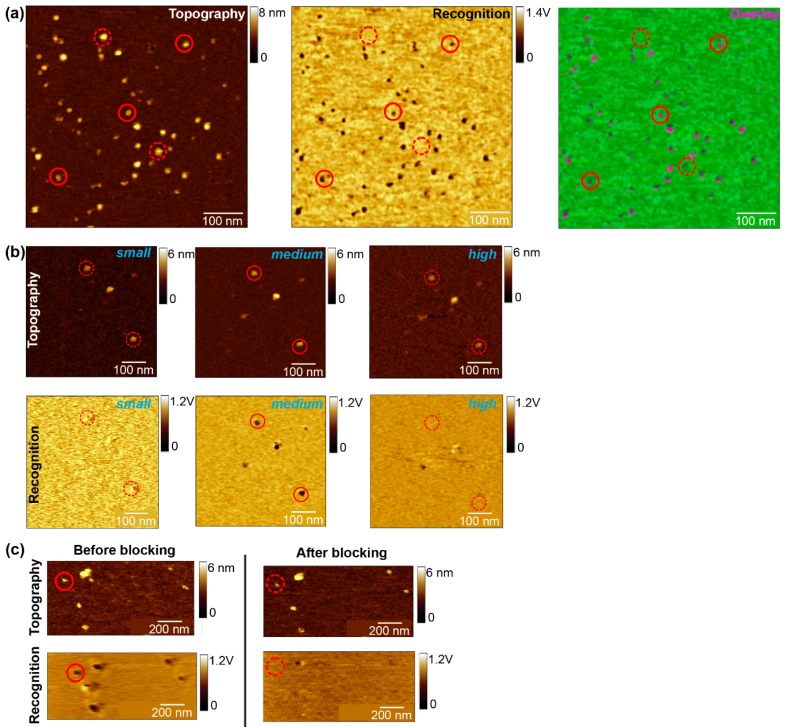
Proof of concept: quantitative characterization of the binding mechanism between Uncoupling Protein 1 (UCP1) and its inhibitor adenosine triphosphate (ATP) (adapted from References [7,10]). First part of the combined experiment, simultaneous topography and recognition imaging to visualize specific interaction between UCP1 and ATP. (**a**) Topography, recognition, and overlay image. UCP1 molecules imaged with an ATP-tethered tip. Black dots in the corresponding recognition image arise from the decrease of the oscillation upwards peaks as a result from UCP1–ATP binding during recognition. The overlay image shows a superposition of the recognition map of UCP1–ATP binding domains (in pink) and the corresponding topography image. Color scale (green to pink) is 0–8 nm. (**b**) Specificity proof of UCP1–ATP interaction by amplitude block experiment. High resolution topographical (top row) and ATP recognition (bottom row) images of UCP1 recorded at different amplitudes. (**c**) Specificity of UCP1–ATP interactions tested by a surface block competition experiment. Topographic and recognition images before blocking of UCP1 (left column) and after UCP1 blocking (right column) by adding free ATP (4 mM) into the solution. (**a**–**c**) Solid and dashed circles indicate recognized and unrecognized protein molecules, respectively. Recognition events are shown in black. Image sizes: 600 × 600 nm.

1. Choose AFM tapping mode in the software.
2. Select the following channels for recognition imaging: height, recognition image channel (commonly referred as AUX channel), amplitude, and phase—both for trace and retrace.
3. Run a tuning curve (0–50 kHz) to set a proper frequency for the acoustic excitation of the cantilever [7]. To achieve an adequate driving amplitude, chose the second frequency level (f ~ 21 kHz) for recognition imaging instead of the base resonance frequency (f ~ 10 kHz) for the MSNL lever.
4. To engage, choose the following settings:
  - Amplitude setpoint ~4 V, adjust with drive.
  - Stop at amplitude reduction of 80%.
5. Start the imaging and optimize the AFM recognition imaging parameters (amplitude setpoint, oscillation frequency, feedback gains). This is partially a trial-and-error process and will differ between the samples, as well as the coupled biomolecule to the tip. Topographs should appear similar regardless of scanning direction (trace and retrace). The corresponding recognition events, displayed in the AUX channel, should match with topographic features. A brief description of some key parameters follows, along with suggested value ranges and can be found in more detail in Preiner et al. [26]:
  - The proper chosen oscillation amplitude is the most prerequisite to achieve reliable recognition events. If the linker is stretched too less at low oscillation amplitudes or, alternatively, if the receptor—ligand bond is broken at each oscillation cycle when using too large amplitudes, no pronounced and stable recognition events are observed. The range of appropriate amplitudes for recognition imaging is determined by the effective linker length (including the position and the length of the molecule attached to the linker), and therefore sharply localized. Run an amplitude–distance curve and test different amplitude setpoints (while keeping the ratio of amplitude setpoint and free amplitude constant) to find the best working amplitude for achieving pronounced recognition signals. The value varies usually between 1.5 and 2 V.
  - Additionally, the variation of the amplitude can be also used as a specificity proof, besides the widely used tip/surface block (Figure 2b) without perturbation of the receptor–ligand system. This procedure is based on the physical property of the signal transduction of the recognition process. Vary the amplitude to very high (~3–4 V) and very low (<1 V) values. If the recognition spots disappear at this value and appear again at the proper set value (~2 V), it will give the user the proof for specific interaction events between the chosen receptor–ligand pair.
  - The oscillation frequency has direct influence on the topographical crosstalk in the recognition signal. Adjust the value always lower than the resonance frequency (2nd order) of the cantilever at tip/surface contact. For lower driving frequencies, the topographical perturbation has more time to decay until the recognition signal is detected.
  - Optimize the feedback gains to allow the AFM tip to accurately track the surface. Increase the gain to the point at which feedback oscillation noise is observed in the AFM topography image. Slightly reduce the gain to remove this effect.
6. Scan the sample with above suggested parameters and find a good sample position, more precisely an area with single proteins incorporated in the lipid membrane and with clearly defined recognition spots which match with the topography. For doing so, first scan a bigger area (~5–10 µm) to find single, big bilayer batches and gradually zoom-in to visualize the proteins in the membrane and to study their recognition with the derivatized molecule on the tip (500–700 nm scan size).
7. **OPTIONAL STEP** Change the sample and/or adjust the incubation time, lipid concentration, temperature, etc., to optimize the formation of single, big lipid bilayer batches on mica with the incorporated molecule homogenously distributed (Figure 2a). If no recognition signals appear after adjusting all parameters, change the tip.
8. After finding a suitable area with a size of 500–700 nm^2^ that covered by a complete membrane batch check whether the membrane proteins are mostly visualized as single, round-shaped structures showing specific interaction (Figure 2a). Then start scanning this area several times up and down without changing any parameters. As soon as no thermal drift occurs anymore, and the subsequent scans show no change in terms of position displacement/shift, stop the measurement and switch from tapping to contact mode.
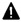
**CRITICAL STEP** Before switching to contact mode, make sure that the thermal drift is as minimal as possible, since the subsequent force spectroscopy experiment is carried out “blindly” without a visual control of the correct cantilever position on the depicted molecule.

#### 3.4.2. Force Spectroscopy: Dynamics and Kinetics of Interacting Partners

1. After changing to contact mode, position the cantilever on a selected recognition spot (functional molecule showing strong binding in TREC mode) using positional feedback control.
2. Start the force spectroscopy experiment by recording force distance curves directly on the depicted spot using well-adjusted parameters (pulling speed, hold times, etc.) (Figure 1c). Since binding/unbinding is a stochastic process record at least 500–1000 curves per pulling speed.
3. **OPTIONAL STEP** In order to probe the dynamics and kinetics of the receptor-ligand interaction vary the pulling speed, and thus, loading rate after recording a data set of 500–1000 curves.
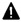
**CRITICAL STEP** Thermal drift may occur during the force spectroscopy experiment and the user will lose the depicted protein while recording force distance curves. This can be recognized from a dramatic drop in the binding probability and no more characteristic binding events in the retraction curve. To readjust the cantilever on the depicted spot, switch back to tapping mode and repeat steps 6 and 8 from Section 3.4.1 to find/trace back the probed molecule. Do not chance the scanning parameters. Start again with steps 1–3.
5. To calibrate the cantilever spring constant apply the thermal noise method according to Hutter et al. [27] at the end of the experiment. Repeat the spring constant determination for each used tip at least five times.
6. Perform a specificity proof, in addition to the amplitude block experiment. After finding a proper scanning area with several recognition spots, inject a surface blocking molecule (~1–10 mM) and scan this area until the recognition spots disappear. Use an excess of blocking molecules (mM range) to guarantee that all the surface molecules are saturated. An almost complete loss of the recognition events after addition of the free molecule, while leaving the topography image unchanged (Figure 2c), will clearly confirm that most of the recognition spots were caused by specific binding of the tip-linked molecule to the proteins in the planar lipid membrane. Select a molecule where the recognition has disappeared and record at least one data set of force distance curves. You should detect almost no binding events with binding probabilities below 5%, due to the specific blocking of the ligands binding pocket in the receptor. Additionally, recording some force distance curves on the pure lipid membrane should also reveal little to no binding events, since specific interaction with the tip-linked ligand and the lipids is not expected. The few remaining recognition/unbinding events can be attributed to unspecific adhesion.

## 4. Expected Results

The described protocol provides a method to simultaneously map and study (single) interaction sites at low lateral density by combining AFM-based recognition imaging and force spectroscopy. The first proof-of-concept was applied to the interaction between UCP1 and ATP [7] for examining the binding and inhibition mechanism of UCP1 and UCP3 by three different types of purine nucleotides (PN): ATP, ADP, and AMP [10]. Moreover, by varying the dynamics of force loading, the binding dynamics was elucidated, and the interaction energy landscape was mapped. As a second example the interaction between SecYEG and SecA was studied. Here we show that this mode is not only useful to extensively study ligand binding on a single molecule level, but also to visualize the orientation of the protein in the membrane [28], as presented here for SecYEG.

### 4.1. Proof-of-Concept: UCP-PN Interaction

In the first step of applying this combined technique, the distribution of the UCP1 molecules in the formed lipid bilayer as well as their functionality in binding ATP was revealed. Mostly single UCP1 molecules were visualized as round-shaped structures in the topography image, (Figure 2a), whereas the black dots visible in the corresponding recognition image reveal the existence of ATP binding sites on UCP1. As the overlay image of both channels indicates, almost all UCPs that were detected in the topography image were recognized by the ATP-functionalized tip. The specificity of the recognition signals was first proven by a so-called amplitude block experiment (Figure 2b), and further by injection of 4 mM free ATP into the buffer solution to competitively block the ATP binding sites in the UCP1 binding pocket (Figure 2c). By varying the amplitude to extreme low or high values, as explained in Section 3.4.1 (step 5), the recognition spots disappeared to appear back again at a proper chosen amplitude. The latter can be clearly seen in Figure 2b, middle panel. After injection of free ATP in solution (Figure 2c), an almost complete loss of recognition spots was observed, while the UCP1 topography remained unchanged. Likewise, we do not see any recognition events on pure lipid membranes. This clearly confirms that most of the recognition spots were caused by specific binding of tip-linked ATP to UCP1 molecules in the planar lipid membrane.

**Figure 3:**
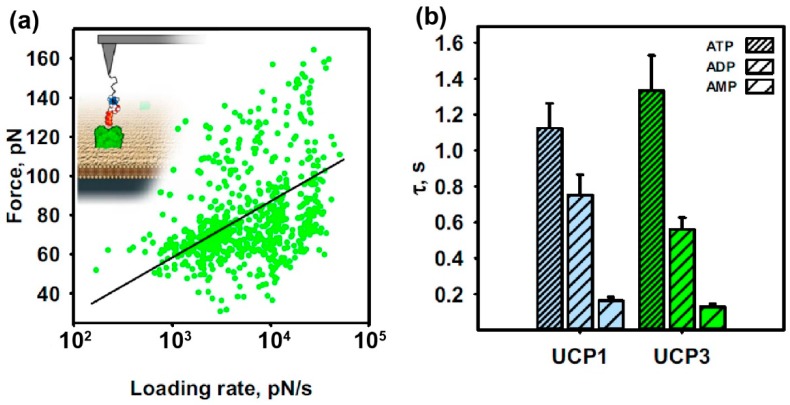
Proof of concept: quantitative characterization of the binding mechanism between UCP1 and its inhibitor ATP (adapted from References [7,10]). Dynamic force spectroscopy to study the binding forces between a pre-selected UCP molecule and its corresponding purine nucleotide (ATP, ADP, AMP). (**a**) Dynamic force spectroscopy plot for the UCP1–ATP interaction. Single-binding events between UCP1 and ATP are shown as green dots. After applying the Bell–Evans model (black line), the bond lifetime can be directly determined. (**b**) Application of this combined mode to other UCP-PN pairs: Dependence of bond lifetimes on PN-phosphorylation for UCP1-PN (blue) and UCP3-PN (green). Shown are the mean values ± SD of at least independent measurements, arising from the fit in (**a**).

After identifying ATP-binding UCP1 molecules, switching from tapping to contact mode and positioning of the cantilever on a selected recognition spot was accomplished. Force spectroscopy experiments were started to probe the binding activity and interaction forces at different pulling speeds. Depending on the applied pulling speed, the binding frequency ranged between 30 and 45% and decreased dramatically to below 5% after injecting a blocking molecule (mM range, excess to guarantee a full saturation of UCP1 molecules) or probing the interaction on a pure lipid membrane lacking the UCP1 molecule. After analyzing the FDCs recorded on UCP1 proteins with respect to unbinding force, loading rate and effective spring constant, a dynamic force spectroscopy (DFS) plot from all the applied pulling speeds was reconstructed. After fitting the data force vs. loading rate data points with the Bell–Evans model [29] (Figure 3a), two important parameters of the interaction energy landscape of UCP1-ATP binding were extracted: the characteristic length scale of the energy barrier, x_β_ ~ 1.65 ± 0.01 Å, and the kinetic off-rate, k_off_ = 0.89 ± 0.11 s^−1^, which is the inverse of the bond lifetime, τ = 1.12 ± 0.14 s.

After the successful proof-of-concept, we further applied this method to study the binding and inhibition of UCP3 (another uncoupling protein) in comparison to UCP1 by different purine nucleotides (Figure 3b). Both proteins are mitochondrial membrane proteins, expressed in different tissues (e.g., brown adipose tissue—BAT), and facilitate proton leakage to support non-shivering thermogenesis. For UCP3, the transport function and the molecular mechanism of the regulation are poorly investigated. Interestingly our data show that the lifetimes of both UCP3-PN (1.33 s, 0.56 s, and 0.13 s for ATP, ADP, and AMP, respectively) and UCP1-PN (1.12 s, 0.75 s, and 0.16 s for ATP, ADP, and AMP, respectively) interactions decrease alongside with the degree of PN-phosphorylation (Figure 3b) [10].

### 4.2. SecYEG

**Figure 4:**
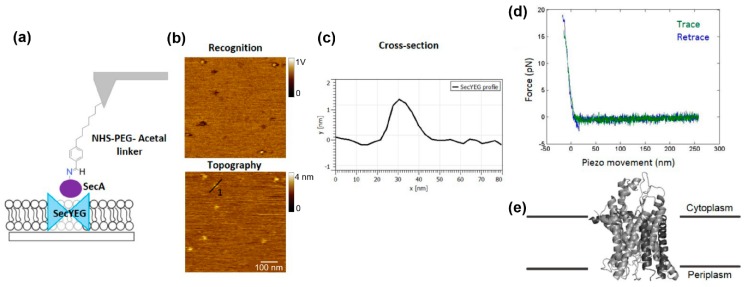
Proof of concept: characterization of the SecA–SecYEG interaction. (**a**) Scheme of SecYEG complexes reconstituted into a planar lipid bilayer with the tip carrying a SecA molecule attached via an N-hydroxysuccinimide (NHS)–PEG–Acetal linker. (**b**) SecYEG molecules that interacted with the SecA on the tip appear as dark spots in the recognition images. (**c**) Cross-section of SecYEG. (**d**) Characteristic force-distance curve for the SecYEG–SecA interaction. (**e**) Side view of the crystal structure of SecYEG from *M. Jannaschii* (PDB-ID 1RHZ).

Visualization of SecYEG molecules on the sample surface was achieved by fusion of SecYEG proteoliposomes on freshly cleaved mica resulting in formation of a planar lipid bilayer. Topographical AFM images showed a low SecYEG density on the sample surface, making the acquisition of accurate and reliable force spectroscopy experiments difficult. By measuring the size of SecYEG molecules on the topographical images, an average height of ~1.2 nm was determined (Figure 4c). This value is in a good agreement with the values reported in the literature [30] for the protrusion of the cytoplasmic site of SecYEG out of the membrane (Figure 4e).

Detection and localization of functional SecYEG molecules at such a low surface density was achieved by employing AFM-based force spectroscopy guided by recognition imaging. An acoustically oscillating AFM tip carrying covalently attached SecA was scanned over the sample surface (Figure 4a). Specific binding of SecA to surface-immobilized SecYEG resulted in generation of a recognition signal (dark spots in Figure 4b). Mapping SecA binding sites by TREC allowed targeting of functional SecYEG channels with nanometer accuracy for force measurements. In a similar manner as thoroughly described in Section 4.1, by switching to force spectroscopy mode (Figure 4d), binding probabilities, unbinding forces, and information on the energy landscape of the interaction between the ATP motor-protein SecA and the translocation channel SecYEG were extracted.

### 4.3. Data Analysis

This step depends on the AFM type and producer used; data analysis tools are usually supplied along with the AFM. Two main types of data analysis need to be carried out. First, the analysis of the AFM topography and recognition images, followed by the analysis of the force curves acquired. In the following, some basic guidelines to analyze and interpret the data are given.

#### 4.3.1. AFM Topography and Recognition Images

1. Load the topography and recognition image into Gwyddion and visualize the in a false-color image (topography: e.g., “golden”; recognition: e.g., “grey scale” or any other color pattern to properly distinguish between topography and adhesion events).
2. Improve the height images by using filters such as flatten or plane fit. 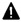 **CRITICAL STEP** Users should keep in mind that application of any filter will—to some extent—modify the raw values and that quantitative parameters should be extracted before use of filters.
3. In order to analyze the height and diameter of the (single) molecules, use a zoom-in image and extract cross-sectional profiles.
4. For allocation of recognition events to topographical features, produce an overlay of those two channels.
5. For comparing different amplitudes at the same scan area (amplitude block) or the images taken before/after the surface block, keep the z-scale value (to adjust the contrast), as well as the threshold (recognition yes or no) always constant. Determine the right threshold from regions without a protein; only when the recognition signal is below this value, consider the protein as to be recognized by the ligand functionalized tip.

#### 4.3.2. Analysis of the Force Curves

For the determination of interaction forces between a specific receptor—ligand pair, force-distance cycles are recorded. The cantilever behaves like a Hookeian spring, where the force F exerted on the sample by the AFM tip scales linearly with the cantilever deflection ∆z, according to Hooke’s law: F = k_c_∆z, k_c_ being the cantilever’s spring constant and z the defection, which should be proportional to the travel distance of the z-piezo in the contact region of the force-distance cycle. The retraction segment of the FD curves is further used for data analysis of unbinding events. To assess the specificity of the interaction, adhesion events must be separated from the contact region of the FD curve. Practically, the rupture distance of a specific adhesion event will depend on the extended length of the PEG linker at the applied force, the size of the molecule attached to the PEG linker and the probed sample. Moreover, by utilizing appropriate biophysical models, the kinetic and thermodynamic parameters of the specific binding events can be extracted to give insight into the binding free-energy landscape.

1. Load the recorded FD-curves into suitable scientific analysis software, such as Matlab (custom-written routines from Keysight commercially available), Gwyddion or PicoView.
2. Depending on the used software, extract the following values:
  - Loading rate from each rupture event: Measure the slope of the force versus distance curve just before the rupture. This gives the value of the effective spring constant k_eff_ (force segment ∆F divided through the distance segment ∆x of the very last part of the rupture event). Multiply this value with the applied pulling speed. Alternatively, as a more elegant way: If force vs. time curves were additionally recorded, the slope of the force vs. time curve just before the rupture event directly results in the loading rate.
  - Rupture force: Take the value on the y-axis (force axis) right on the (negative) maximum peak of the rupture event before it returns to baseline level. In case of curves containing multiple rupture events, each rupture should be considered separately.
  - Plot the extracted unbinding force versus the loading rate on a semi-logarithmic scale (DFS plot) using Origin or a similar software, which allows to extract kinetic and thermodynamic parameters using appropriate models and strategies [31].
  - If desired, make unbinding force histograms or probability density functions (pdfs) from separated, distinct loading rate ranges to capture all the information contained in the DFS plot, such as the presence of multiple interactions. Fit the histograms with Gaussian distributions to determine the presence of single or multiple peaks, directly corresponding to one or several receptor–ligand interaction(s). The advantage of using pdfs instead of histograms is that data binning is avoided and the data will be analyzed more precisely, since the values are weighted to their reliability [32].
  - Fit the loading-rate-dependent interaction forces with the fitting model of choice. Such models can be a linear iterative fitting algorithm (Levenberg Marquardt) along with the Bell–Evans model [29] (Figure 3a) or a non-linear iterative fitting algorithm (Levenberg Marquardt) along with the Friddle–Noy–de-Yoreo (FNdY) model [33]. The simplest and most commonly used model is the Bell–Evans fit for crossing a single energy barrier, where the user is obliged just to take single interactions into account. Here, exclude multiple binding events from the force pdfs, which appear as a “shoulder” in the Gaussian distribution of the most probable unbinding force. Exclude these data and use only data within the interval [μ ± σ] for construction of the DFS plots and the subsequent fitting with the Bell–Evans model.
  - An extensive overview of analysis and fitting of dynamic force spectroscopy (DFS) data is given by Bizzarri and Cannistraro [1], as well as by Noy and Friddle [33], whereas Hane and colleagues have compared the dominant models in great detail [34]. The choice of model for data analysis relies first and foremost on the obtained force spectrum; non-linear alternatives to the Bell–Evans model should be used only if the forces measured do not scale linearly with the logarithm of the loading rate—as fitting a linear dependence with a non-linear function may result in fit parameters with little to no meaning.

## 5. Reagents Setup

- Assay buffer.
  ○ UCP1–ATP example: 50 mM NaSO_4_, 10 mM MES, 10 mM Tris, 0.6 mM EDTA, adjusted to pH 7.2.
  ○ SecYEG–SecA example: 50 mM Tris, 50 mM NaCl, 50 mM KCl, 5 mM MgCl_2_, adjusted to pH 7.0.
- Citric acid 1% (*w*/*v*). Dissolve 0.5 g of citric acid in 50 mL of Milli-Q H_2_O. Store at −20 °C in 2-mL aliquots for up to 2 years.
- Ethanolamine solution (1 M, pH 8.0). Dissolve 1.2 g of ethanolamine in 20 mL of Milli-Q H_2_O. Adjust the pH to 8.0 with 1 M HCl. Store at −25 °C in 0.5-mL aliquots for up to 2 years.
- Sodium cyanoborohydride (1 M solution with 20 mM NaOH). Working under a chemical hood, place 13 mg of NaCNBH_3_ into a polyethylene capped glass weighing bottle. Pipette 20 μL of 100 mM NaOH on top of the NaCNBH_3_ and mix well by pipetting. Then add 180 μL of Milli-Q water and mix by pipetting. If the balance used for weighing is outside of the chemical hood, first zero the balance using the empty weighing bottle with lid on, and then add ~13 mg of NaCNBH_3_ to the bottle under the hood. Cap the bottle and weigh. Adjust volumes of NaOH and Milli-Q water proportionately. 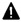 **CAUTION** NaCNBH_3_ is highly toxic. Wear gloves and handle with care. Handle under a chemical hood during preparation. 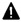 **CAUTION** Prepare fresh NaCNBH_3_ for each functionalization. Dispose of any remaining NaCNBH_3_ according to institutional regulations.
